# Preparation of Fecal Microbiota Transplantation Products for Companion Animals

**DOI:** 10.1371/journal.pone.0319161

**Published:** 2025-04-09

**Authors:** Nina K. Randolph, Matthew Salerno, Hannah Klein, Dubraska Diaz-Campos, Joany C. van Balen, Jenessa A. Winston

**Affiliations:** 1 Department of Veterinary Clinical Sciences. College of Veterinary Medicine, The Ohio State University. Columbus, Ohio, United States of America; 2 Comparative Hepatobiliary and Intestinal Research Program, College of Veterinary Medicine, The Ohio State University, Columbus, Ohio, United States of America; Washington State University - Spokane, UNITED STATES OF AMERICA

## Abstract

Fecal microbiota transplantation (FMT) is increasingly utilized in small animal medicine for the treatment of a variety of gastrointestinal and non-gastrointestinal disorders. Despite proven clinical efficacy, there is no detailed protocol available for the preparation and storage of FMT products for veterinarians in a variety of clinical settings. Herein, the effect of processing technique on the microbial community structure was assessed with amplicon sequence analysis. Microbial viability was assessed with standard culture techniques using selective media. Given the fastidious nature of many intestinal microbes, colony forming units are considered surrogate viable microbes, representing a portion of potentially viable microbes. FMT products from four screened canine fecal donors and six screened feline fecal donors were processed aerobically according to a double centrifugation protocol adapted from the human medical literature. Fresh feces from an additional three screened canine fecal donors were used to evaluate the effect of cryopreservative, centrifugation, and short-term storage on microbial community structure and *in vitro* surrogate bacterial viability. Finally, fresh feces from a third group of three screened canine and three screened feline fecal donors were used to evaluate the long-term *in vitro* surrogate bacterial viability of three frozen and lyophilized FMT products. Microbiota analysis revealed that each canine fecal donor has a unique microbial profile. Processing of canine and feline feces for FMT does not significantly alter the overall microbial community structure. The addition of cryopreservatives and lyopreservatives significantly improved long-term viability, up to 6 months, for frozen and lyophilized FMT products compared to unprocessed raw feces with no cryopreservative. These results prove the practicality of this approach for FMT preparation in veterinary medicine and provide a detailed protocol for researchers and companion animal practitioners. Future *in vivo* research is needed to evaluate how the preparation and microbial viability of FMT impacts the recipient’s microbial community and clinical outcomes across multiple disease phenotypes.

## 1. Introduction

The intestinal microbiome is a complex ecosystem composed of trillions of diverse microbes including bacteria, archaea, protozoa, fungi, and viruses. The composition of the gut microbiota is unique to each individual and is shaped by multiple factors including diet, age, host genetics, environment, lifestyle, and medical history [1]. Mammalian health and homeostasis are dependent on a robust and diverse intestinal ecosystem and its myriad of metabolic activities [2,3]. Bacteria are the most abundant constituent of the intestinal microbiome, accounting for approximately 80-90% of all microbes [4]. These microbes are metabolically active, have a direct impact on host physiology, metabolism, and immune function; thus, collectively the gut microbiota acts as an organ by directly contributing to the health status of the host [5]. An ever-expanding number of gastrointestinal (GI) and non-GI diseases are associated with dysbiosis, an imbalance in microbial community structure and function[2]. Certain medications, particularly antimicrobials, are implicated as a cause of severe and long-lasting alterations in the gut microbial ecosystem which may be detrimental to the host [6–8]. Our rapidly growing knowledge of dysbiosis and its implications on companion animal health have led to intense interest in how to rationally manipulate the gut microbiome to restore eubiosis and promote microbiome health with the ultimate goal of improving patient outcomes.

Fecal microbiota transplantation (FMT) is the transfer of feces from a healthy donor into the gastrointestinal tract of a diseased recipient with the intent to modulate the recipient’s microbiome and confer a health benefit. The precise mechanism in which FMT confers a health benefit is incompletely understood, but is likely disease specific and linked to the viability of microbes, microbial engraftment within the recipient, and ability to restore eubiosis in the intestinal ecosystem [9].

Techniques for FMT production in human medicine vary across studies, commercial fecal banks, and laboratories. Similar to veterinary hospitals, these differences are in part due to a variety of factors that vary between institutions that offer FMT therapy [10]. Access to equipment such as -80°C freezers, lyophilizers, anaerobic chambers, and large centrifuges may dictate protocols for processing and storage of FMT products in a clinical setting.

As FMT is used more frequently in human and veterinary clinical practice, convenient storage and administration has become important for clinicians and patients alike. The addition of 10% glycerol is common practice across human stool banks when freezing FMT products in order to increase shelf-life and improve overall microbial viability [11–15]. Lyophilization, also known as freeze-drying, is the process of removing moisture through sublimation at low temperature and pressure conditions. The final product, known as a “cake”, can be ground to a fine powder and transferred into capsules for oral FMT administration [16]. Lyophilized product remains biologically active [17] and is capable of inducing remission of recurrent *C. difficile* infection and ulcerative colitis in human patients [18–20]. To date, only a single peer-reviewed pilot study has evaluated the impact of the commercially available AnimalBiome® lyophilized FMT capsules on the canine microbiome [21]. In this pilot study, three dogs with chronic diarrhea were administered twice daily AnimalBiome® capsules for 60 days, which resulted in persistent improvement of fecal consistency and shifts in several microbial families for up to 60 days following cessation of daily FMT capsular treatment [21].

In veterinary medicine, each facility preparing FMT products uses different processing and storage techniques, which may contribute to: inconsistencies in microbial composition and viability, variability in clinical efficacy, and ultimately patient outcomes. Therefore, the goal of this project was to propose standardized fecal processing techniques to provide companion animals with access to safe and effective FMT products aimed at maximizing bacterial viability and establishing shelf-life recommendations. These protocols for FMT production, the first of their kind, are intended for processing canine and feline feces and provides veterinarians with evidence for producing and storing FMT products in their own practice based on their availability of resources and equipment.

## 2. Materials and Methods

**Fig 1:**
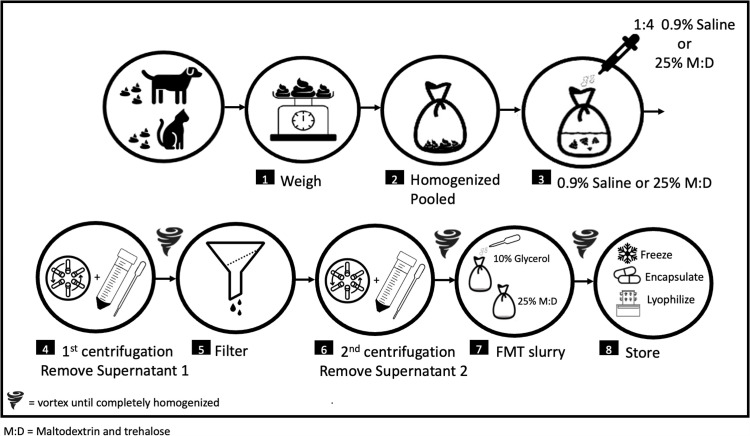
Schematic illustration of FMT processing with double centrifugation.

The protocol described in this peer-reviewed article is published on protocols.io, **dx.doi.org/10.17504/protocols.io.3byl493njgo5/v1** and is included for printing as supplemental file 1 with this article. Overall procedures are outlined in Fig 1.

### 2.1 Animals and experimental design

Naturally voided feces from client-owned screened canine and feline fecal donors from the OSU Companion Animal Fecal Bank (CAFB) were used for this study (Table 1). A detailed description of the donor screening protocol is outside the scope of this manuscript. All donors were screened in accordance with guidelines put forth by the Companion Animal FMT Consortium[22], Readers are invited to review the donor screening protocols described therein. All protocols were approved by The Ohio State University International Care and Use Committee (IACUC number 2017A00000093-R1).

**Table 1:** Demographics of seven screened canine fecal donors and six screened feline fecal donors. For feline fecal donors, all but two donors are from multiple cat households. Abbreviations: Fel; feline; K9; canine; MC; male castrated; FS; female spayed; M; male.

Fecal Donor #	Age (years)	Sex	Breed
K9 1	3	M	Munsterlander Pointer
K9 2	2	M	Belgian Malinois
K9 3	2	FS	Mixed Breed Dog
K9 4	3	MC	Mixed Breed Dog
K9 5	1.5	M	Cavalier King Charles Spaniel
K9 6	6	FS	Siberian Husky
K9 7	1	MC	Mixed Breed Dog
Fel 1	5.5; 4	FS; FS	Domestic Shorthair (n = 2)
Fel 2	3.5; 3.5	FS; FS	Domestic Shorthair (n = 2)
Fel 3	2.5	MC	Domestic Medium Hair
Fel 4	4.5; 4; 3	FS; FS; MC	Domestic Shorthair (n = 3)
Fel 5	3.5	MC	Domestic Shorthair
Fel 6	6; 3	MC; MC	Domestic Shorthair (n = 2)

**Evaluating the impact of fecal processing and centrifugation**

FMT products from two groups of canine donors were evaluated. The first group (donors 1-4) were enrolled during the height of the SARS-CoV-2 pandemic. Strict COVID-19 protocols temporarily precluded frequent travel of owners to the university laboratory, necessitating a deviation in FMT processing protocol. During this time, owners stored donor feces in their home -20°C freezer until a gallon freezer bag was filled. Frozen feces were brought to the laboratory in large batches and were placed in a -80°C freezer upon receipt. Feces were thawed on ice overnight, then processed using the double centrifugation technique with 10% glycerol (outlined in 2.1). Multiple batches were prepared from donors 1-4; 5, 6, 7, and 3 batches respectively. Two hundred and fifty microliter aliquots were taken from each processing step for DNA extraction and amplicon sequencing (V4 region of 16S rRNA gene). Samples included the homogenized pooled unprocessed feces, saline mixture, and final fecal slurry with glycerol (Fig 1; Steps 2, 3, and 7 respectively).

**Evaluating the impact of cryopreservative and storage conditions**

To determine the effect of cryopreservatives and storage conditions on microbial composition and bacterial viability, a second group of canine donors (donors 5-7) each provided one fresh fecal sample. Feces were refrigerated within 15 minutes of defecation and brought to the laboratory on ice within 2 hours of defecation. Fecal processing techniques included unprocessed (raw), 0.9% saline with 10% glycerol, and two double centrifuged fecal slurries with the following additives: 0.9% saline with 10% glycerol, and 0.9% saline with 25% maltodextrin and trehalose (M:D) (Fig 1; Steps 2, 3, and 7 respectively). Colony forming units (CFUs) were enumerated immediately after processing and lyophilization (baseline) and at two weeks of frozen storage at -80°C.

To determine the impact of fecal processing on feline microbial composition, two batches of fecal slurry were produced from two separate pooled and homogenized batches of feces from 4 screened feline fecal donors. A single batch of feces was provided from two additional screened feline fecal donors. Amplicon sequencing (V4 region of 16S rRNA gene) was performed on homogenized pooled feces and the final 10% glycerol FMT slurry (Fig 1: Steps 2 and 7).

To determine the effect of cryopreservatives and long-term storage on selected bacterial viability, three additional screened canine and three screened feline fecal donors each provided three fresh fecal samples. Lyophilized and frozen slurry were cultured at baseline and at 6 months of storage. FMT slurry was stored at -80°C and lyophilized products were stored at room temperature.

### 2.2 DNA extraction and amplicon sequencing

Amplicon sequencing was used to determine the impact of fecal processing on microbial community structure. All samples were sent to the University of Michigan Microbiome Core for DNA extraction and amplicon sequencing. DNA was extracted from FMT products using a MagAttract PowerMicrobiome kit (Qiagen, Germantown, MD, USA) following manufacturer instructions. Sequencing of the V4 region of the 16S rRNA gene amplicon was performed using a dual indexing approach as previously described[23,24]. Briefly, universal primers 515f and 806r were used for gene amplification [25]. DNA products were initially incubated at 97°C for 120 seconds; followed by 30 PCR amplification cycles. Denaturation occurred at 95°C for 20 seconds, annealing at 55°C for 15 seconds, then 72°C for 900 seconds. PCR products were maintained at 4°C until product visualization with SYBR Safe DNA Gel Stain (ThermoFisher Scientific). A SequalPrep Normalization Plate Kit (Life technologies, Carlsbad, CA, USA) was utilized for amplicon library normalization. A Kapa Biosystems Library Quantification kit for Illumina platforms (Kapa Biosystems, Wilmington, MA, USA) was used to determine the final concentration of pooled samples. Each sample was normalized to the lowest sample concentration.

Amplicon sequencing was performed using an Illumina MiSeq platform with a MiSeq reagent kit (Illumina, San Francisco, CA, USA) using V2 chemistry with 500 cycles as previously described [24]. FASTQ files were generated for each paired-end read. Master mix and sterile extraction reagents served as negative controls throughout DNA processing, amplification, and sequencing procedures.

### 2.3 Selected surrogate bacterial viability of FMT products

In order to capture a generalized representation of potentially viable microbes, culture-dependent techniques were employed under aerobic and anerobic conditions to evaluate the short-term and long-term (6 months) viability of microbes in frozen and lyophilized FMT products. Due to the specific growth requirements of many intestinal microbes, only a small portion can be isolated with standard culture methods. As a result, the colonies cultured in this study act as surrogate viable microbes, representing only a portion of the overall microbial community. Aliquots of frozen FMT products were rapidly thawed for 15 minutes in a 37°C water bath, then plated directly and serially diluted with sterile 1X phosphate-buffered saline (PBS). Aliquots of lyophilized FMT product was diluted to a 1:10 stock solution, then serially diluted. Serial dilutions were performed under aerobic conditions. Under aerobic and anaerobic conditions, dilutions were inoculated onto MacConkey agar (Becton Dickinson Biosciences [BD] Difco^TM^) using the track plating technique as previously described [26] and on Columbia colistin naladixic acid (CNA) agar with 5% sheep blood (Remel, ThermoFisher Scientific) using the spread plate technique [27]. MacConkey and CNA agar are selective media for Gram-negative and Gram-positive organisms, respectively. Plates were incubated at 37°C, and CFUs/gram feces/FMT product were enumerated after 48 hours. Anaerobic plating and incubation were performed in a vinyl anaerobic chamber at 0-5 ppm oxygen and 2.0 to 2.5% hydrogen concentration (Coy Laboratory Products, Grass Lake MI). Culture experiments from each fecal sample were performed on separate days and were plated in duplicate each day. After enumeration, to identify viable bacteria growing on selective agar, 200 representative colonies were collected: 100 from aerobically incubated agar and 100 from anaerobically incubated agar. Colonies were scraped off of the agar using a sterile inoculating loop and placed into a sterile 2 mL epitube containing 500 microliters sterile 1X PBS. Epitubes containing aerobic and anaerobically grown colonies were combined and submitted for amplicon sequencing as described in section 2.2.

### 2.4 Statistical analysis

**Microbial analysis**

All fastq files were evaluated for sequence quality and read depth using the FastQC software program (Babraham Bioinformatics*,* version 0.11.9) [28]. Sequences were imported into R Studio and paired-end reads were assembled into contigs, trimmed, filtered, and converted into amplicon sequence variants (ASVs) using the DADA2 pipeline as previously described [29]. Chimera sequences and ASVs < 250 base pairs or > 256 base pairs in length were removed. Sequence classification was performed using the Silva 16S rRNA Sequence Database (Version 138.1) [30,31]. ASVs comprising less than 1% of the total sequences in each sample were removed from composition bar plots and listed as “other” for data visualization and descriptive statistics. Alpha and beta diversity analysis were performed using the phyloseq (Version 1.40.0) and vegan (Version 2.6-2) packages in R studio (Version 2022.07.1, Vienna, Austria). Alpha diversity metrics were calculated using the Shannon Diversity Index and Mann-Whitney was used for significance testing. Beta diversity was calculated using the Bray-Curtis dissimilarity algorithm with significance testing by Permutational Analysis of Variance (PERMANOVA) and visualized with principal coordinate analysis. Significance was defined as *p < 0.05.* All statistical analysis was performed using R software and GraphPad Prism (Version 9.4.0, GraphPad Software, LLC, La Jolla, CA, USA).

**Bacterial viability**

For each FMT product type, absolute bacterial counts (CFU/g) were calculated by totaling CFUs/mL from each dilution/plate. A Shapiro-Wilk test for normality determined a nonparametric distribution of total CFUs/mL. Kruskal-Wallis testing was used to assess the impact of cryopreservatives and storage time on total CFUs/mL for each FMT product. Significance was defined as *p < 0.05.*

## 3.0 Results

### Evaluating the mpact of fecal processing on microbial community structure

**Fig 2:**
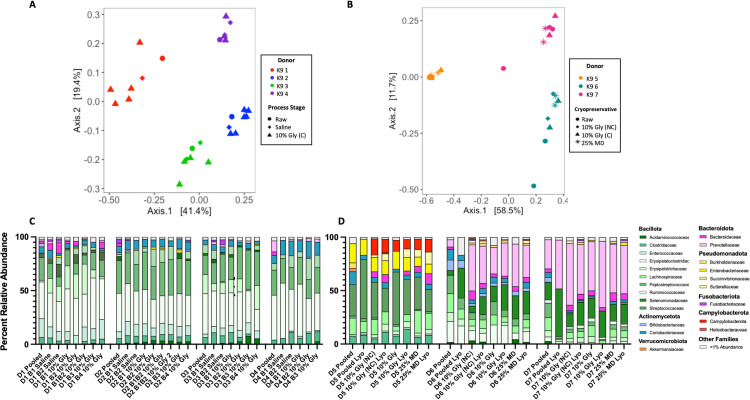
Fecal microbiota composition clusters by individual canine donors, regardless of cryopreservative or processing technique. A/B) Principal coordinate analysis (PCoA) ordination was calculated with Bray-Curtis dissimilarity of ASVs from feces/FMT product. Statistical significance of microbial community structure between canine donors and within donors was determined with PERMANOVA testing with Bray-Curtis dissimilarity. Each canine donor has a distinct microbial community structure compared to all other donors (PERMANOVA: donors 1-4, *p < 0.01*; donors 5-7, *p < 0.001*). C) Family relative abundance of unprocessed pooled (raw) feces, saline mixture, and final 10% glycerol FMT products. D) Family relative abundance of unprocessed pooled feces and 3 frozen and lyophilized FMT products. Abbreviations: B, Batch; C, centrifuged; Gly, gylcerol; K9; Canine; NC, not centrifuged; MD, maltodextrin and trehalose.

**Fig 3:**
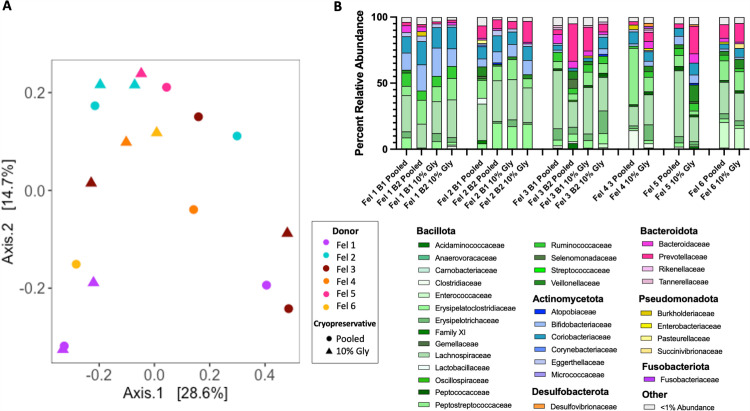
Fecal microbiota composition does not consistently cluster by individual feline households. A) Principal coordinate analysis (PCoA) ordination was calculated with Bray-Curtis dissimilarity on ASVs from feces/FMT product. Pairwise PERMANOVA testing revealed each pair of donors did not have significantly different microbial compositions (p. adj ≧ 0.2 for all donor pairs). In feline donors 1, 2, and 3, there was no significant difference between pooled and final FMT products (feline 1, *p* = 1.00; feline 2, *p* = 0.67; feline 4, *p* = 1.00). A single pooled and 10% glycerol sample precluded PERMANOVA testing in felines 4, 5, and 6. B) Family relative abundance of unprocessed pooled (raw) feces and final 10% glycerol FMT products. Abbreviations: B, Batch; Fel; feline; Gly, gylcerol.

Overall, processing of feces into FMT products does not significantly alter the overall microbial community structure in dogs (Fig 2A and 2B) or cats (Fig 3A). For canine FMT products, beta diversity analysis revealed that regardless of processing step, FMT products clustered by individual canine donors (Figs 2C and 2D, Table 2). In contrast, feline FMT products did not cluster by household (Fig 3A, Table 2). Within individual canine and feline fecal donors, overall microbial community structures between unprocessed (raw) pooled feces and the final processed FMT product were not significantly different (Fig 2A and 2B; canine donors 1-4 PERMANOVA *p =* 0.167 – 1.0; canine donors 5-7, PERMANOVA *p* = 0.128 – 0.365; Fig 3A; feline donors 1, 2, and 3, PERMANOVA *p* = 0.67 – 1.00). Further, lyophilization did not significantly alter microbial community structure when compared to frozen feces/FMT (canine donor 5 PERMANOVA *p = *0.57; canine donor 6 PERMANOVA *p* =  0.59; canine donor 7 PERMANOVA *p* =  0.07).

**Table 2:** The microbial community structure of canine and feline FMT products is not significantly changed following a standardized fecal processing protocol. In these analyses of 7 dogs and 3 cats, pooled unprocessed (raw) feces and the final FMT product were compared using the PERMANOVA significance test. Significance was defined as *p* < 0.05 for all analyses. Abbreviations: Fel; feline; K9; canine.

^Pooled vs Final^	^Adjusted p^
^K9 1^	^0.166^
^K9 2^	^0.136^
^K9 3^	^1.000^
^K9 4^	^0.25^
^K9 5^	^0.365^
^K9 6^	^0.128^
^K9 7^	^0.15^
^Fel 1^	^1.00^
^Fel 2^	^0.67^
^Fel 3^	^1.00^

In all canine pooled unprocessed (raw) feces, Bacillota were the predominant phylum, comprising 58.32 to 94.02% of raw feces (Fig 2C and 2D). Actinomycetota was the second most abundant phylum in pooled unprocessed (raw) feces from canine fecal donors 1, 2, 3, and 6, comprising 4.77 to 20.20% of unprocessed samples. Bacteroidota was the second most abundant phylum in the pooled unprocessed (raw) feces of canine fecal donor 4 and 7 at 13.01% and 30.8%, respectively. Finally, Pseudomonadota was the second most abundant phyla in donor 5 and made up 23.63% of bacterial phyla (Fig 2C and 2D).

The composition of microbial phyla in cats was similar to those observed in dogs. In all feline pooled unprocessed (raw) feces, Bacillota was the predominant phyla, comprising 48.65 to 81.43% of raw feces (Fig 3). Actinomycetota was the second most abundant phylum in raw feces from feline donors 1, 2, 3, and 5, comprising 3.12 to 28.75% of raw feces. Bacteroidota was the second most abundant phylum in feline donors 4 and 6, and comprised 2.98 to 33.9% of raw feline feces.

**Fig 4:**
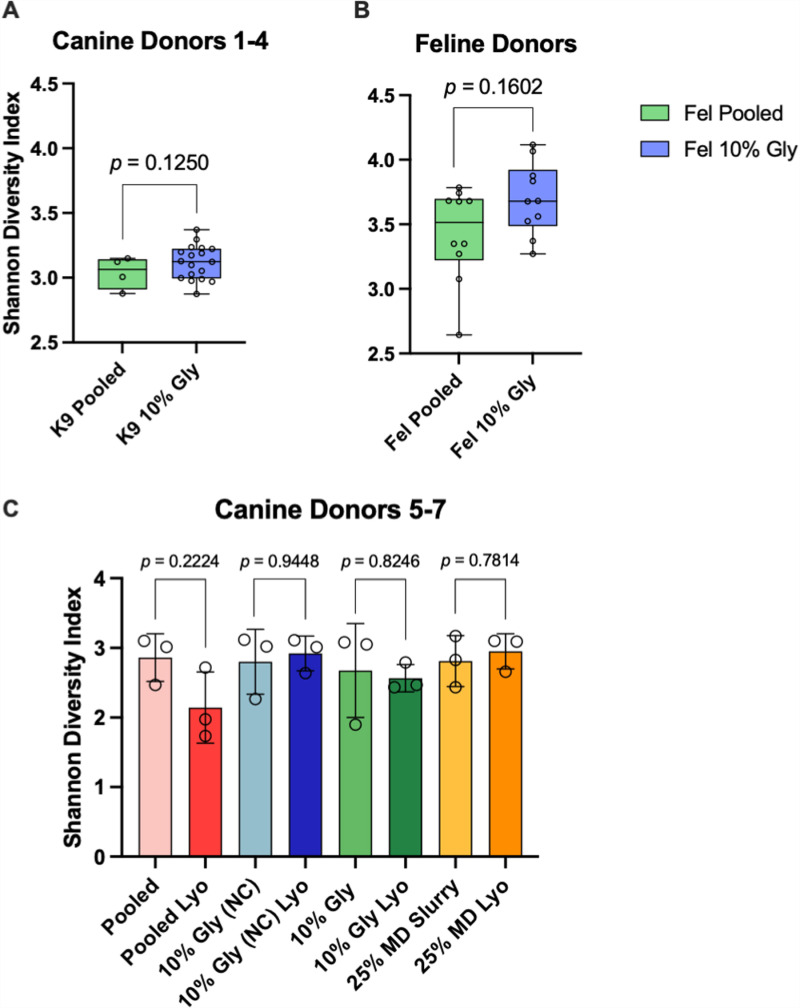
Fecal processing and lyophilization does not significantly impact alpha diversity (Shannon index). A,B) Double centrifugation and the additional of 10% glycerol does not significantly impact alpha diversity in canine and feline FMT products (canine, *p* = 0.3420; feline, *p* = 0.7344). C) Lyophilization does not significantly impact alpha diversity across all FMT products, however FMT products without any cryopreservative exhibited the greatest change between pre- and post-lyophilization. There was no significant difference in Shannon index between unprocessed and processed FMT products (dogs, *p* > 0.1250; cats, *p* = 0.1602). Significance testing was performed using the Mann-Whitney test and significance was defined as *p* < 0.05 for all pairwise analyses. Open circles represent individual samples. Abbreviations: Gly, gylcerol; Lyo, lyophilized; NC, not centrifuged; MD, maltodextrin and trehalose.

Fecal processing did not significantly impact alpha diversity in all canine and feline FMT products (Fig 4; Mann-Whitney test; canine donors 1-4, *p* = 0.342; canine donors 5-7, *p* > 0.999; feline donors 1-6, *p* = 0.734). Lyophilization of canine feces did not significantly impact alpha diversity (Mann-Whitney test; *p* = 0.22 – 0.94) (Fig 4). The alpha diversity (Shannon Index) of feces lyophilized without any cryopreservative exhibited a median reduction of 1.04, while all other FMT products had a change of less than 0.2, but this did not reach statistical significance (*p* = 0.22).

**Evaluating the impact of centrifugation on surrogate bacterial viability**

Using multiple types of selective agar to assess bacterial viability has been applied to human FMT products [13,15,32] and colonies observed on these media are considered surrogates for the entire gut microbial community. Assessment of surrogate bacterial viability was performed herein using CNA and MacConkey agar incubated in aerobic and anaerobic environments. These media are selective for Gram-positive and Gram-negative bacteria, respectively.

**Fig 5:**
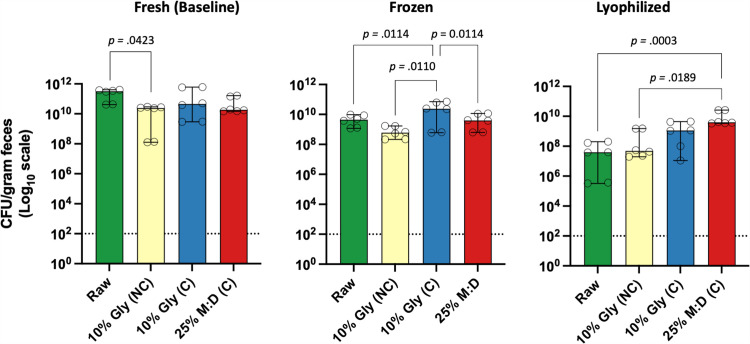
The *in vitro* surrogate bacterial viability of four fresh, frozen, and lyophilized canine FMT products. After freezing, the centrifuged 10% glycerol FMT product did not exhibit a significant loss in surrogate bacterial viability and had significantly more CFU/g than all other FMT products. Open circles represent individual replicates. The dashed line represented the limit of detection for bacterial growth using culture-based techniques. Following normality testing, significance testing was performed using one way ANOVA or Kruskal-Wallis test with the two-stage linear step-up procedure of Benjamini, Krieger, and Yekutieli, as appropriate. Significance was defined as p < 0.05 for all pairwise analyses. Pairwise FMT products with statistically significant differences in total CFU/g are shown. Abbreviations: C, centrifuged; Gly, gylcerol; NC, not centrifuged; MD, maltodextrin and trehalose.

Three screened canine fecal donors (donors 5 – 7) each provided feces from a single defecation. Processing was performed within 2 hours of defecation and four techniques were used: unprocessed (raw), 10% glycerol with no centrifugation (denoted as “NC”), 10% glycerol with double centrifugation, and 25% M:D with double centrifugation (denoted as “C”). The concentration of surrogate viable bacteria (CFUs/gram) in the centrifuged frozen 10% glycerol FMT product was significantly higher than all other FMT products and exhibits a lower log_10_ drop in viability after freezing (Fig 5 and Table 3). Raw FMT products exhibited a statistically significant decrease in total CFUs/gram after lyophilization and two weeks of storage at -80°C (Table 3). Canine frozen M:D FMT exhibited a statistically significant decrease in CFUs/gram after two weeks of frozen storage (Fig 5, Table 3). Lyophilized FMT with 25% M:D exhibited the best maintenance of viability compared to all other lyophilized FMT products (Fig 5 and Table 3). Interestingly, 25% M:D is the only preservative that exhibited a greater loss of viability after freezing than after lyophilization.

**Table 3:** Centrifuged FMT products consistently exhibit the lowest median log_10_ drop of CFU/g following freezing and lyophilization. In contrast, unprocessed feces exhibited the greatest median log_10_ drop and had a statistically significant loss of bacterial viability across both storage conditions. Products with M:D were the only cryopreserved products to exhibit a significant decrease in surrogate bacterial viability after freezing. However, M:D FMT products exhibited the smallest median loss of bacterial viability following lyophilization and outperforms other lyopreservatives. Data is represented as median and range in parentheses. Significant p-values are bolded. Abbreviations: C, centrifuged; NC, not centrifuged; MD, maltodextrin and trehalose.

Product Type	Baseline Fresh Log_10_ CFU/g	Frozen Log_10_ CFU/g	Log drop after 2 weeks of storage at -80C	Lyophilized Log_10_ CFU/g	Log drop following lyophilization	Wilcoxon Hypothesis Test p-values
Unprocessed	11.50 (10.61, 11.61)	9.64 (9.08, 9.99)	-1.54 (-2.49, -0.93)	7.59 (5.52, 8.30)	-3.33 (-6.02, -3.02)	Baseline vs -80: ***0.03*** Baseline vs Lyo: ***0.03***
10% Glycerol (NC)	10.39 (8.10, 10.47)	8.78 (8.34, 9.99)	-1.16 (-1.73, 0.24)	7.68 (7.29, 9.18)	-2.70 (-3.18, 1.06)	Baseline vs -80: 0.16 Baseline vs Lyo: 0.16
10% Glycerol (C)	10.64 (9.46, 11.77)	10.37 (8.78, 10.86)	-0.66 (-0.98, -0.22)	9.05 (7.04, 9.64)	-1.59 (-4.71, 0.18)	Baseline vs -80: 0.39 Baseline vs Lyo: 0.16
25% MD (C)	10.26 (10.16, 11.22)	9.60 (8.80, 10.06)	-1.13 (-1.37, -0.66)	9.60 (9.53, 10.42)	-0.63 (-1.64, 0.18)	Baseline vs -80: ***0.03*** Baseline vs Lyo: 0.16

**Fig 6:**
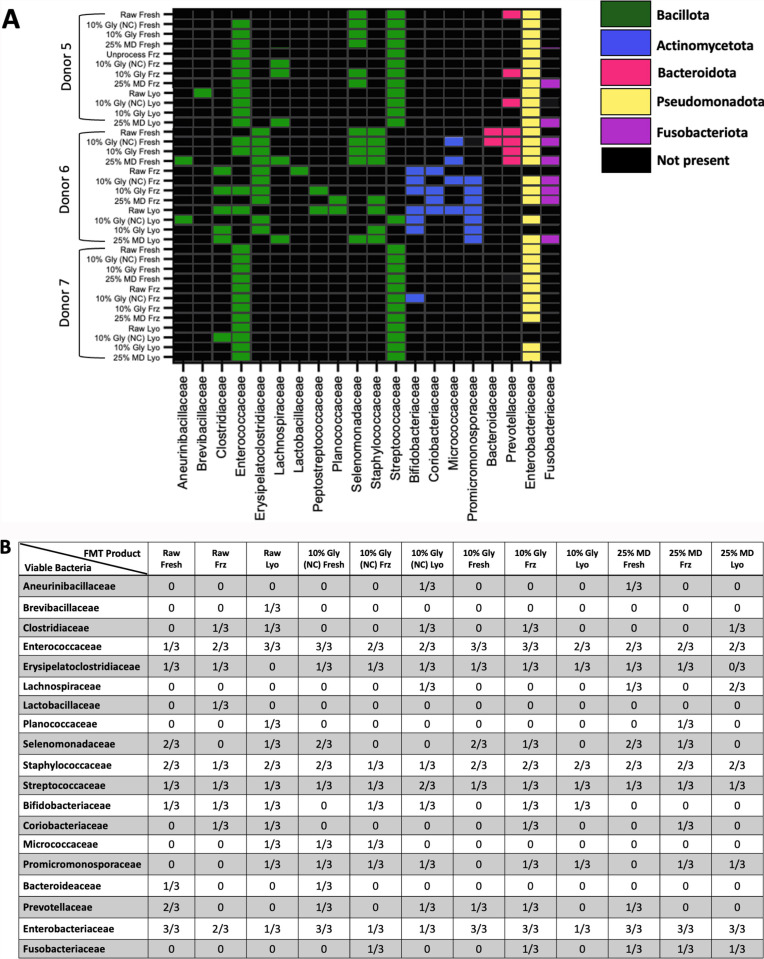
The identity of viable surrogate bacteria grown using selective agar in aerobic and anaerobic environments from canine FMT products. The viability of bacteria varies across canine donor and FMT product type. A) Green, blue, yellow, red, and purple cells indicate the presence of their respective phyla as colonies on agar plates. Black boxes indicate no colonies were isolated from that FMT product. B) Chart totaling each surrogate bacteria grown based on FMT product based on total number of samples submitted for amplicon sequencing. Abbreviations: Gly, gylcerol; Frz, frozen; NC, not centrifuged; MD, maltodextrin and trehalose; Lyo; lyophilized.

To define which surrogate bacteria grew colonies during viability testing, amplicon sequencing was performed on colonies isolated from agar plates of all FMT products from canine donors 5-7 (Fig 6). *Enterobacteriaceae* was viable in all fresh FMT products. However, *Enterobacteriaceae* was not detected in two out of three donors in raw lyophilized, raw frozen, 10% glycerol (NC) lyophilized, 10% glycerol (NC) frozen, and 10% glycerol lyophilized (Fig 6). This finding suggests that *Enterobacteriaceae* is more susceptible to lyophilization and freezing than other microbes. In canine donor 5, *Streptococcaceae* were viable in all FMT products regardless of storage condition. Canine donor 6 was the only donor with viable microbes in the *Actinomycetota,* *Bacteroidota,* and *Fusobacteriota* families. *Actinomyceotota* growth was primarily observed in frozen and lyophilized products. *Fusobacteriota* growth was observed in fresh, frozen, and lyophilized 25% M:D products and frozen 10% glycerol products. In canine donor 6, *Enterococcaceae* and *Streptococcaceae* were viable in all FMT products, regardless of storage condition.

**Evaluating the impact of cryopreservative and long-term storage conditions surrogate bacterial viability**

**Fig 7:**
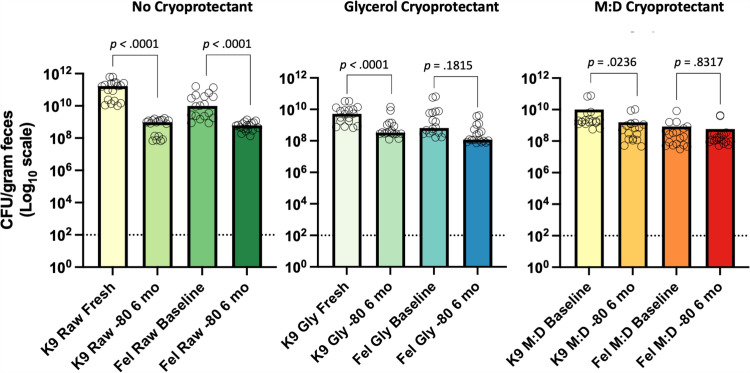
The long-term *in vitro* surrogate bacterial viability of canine and feline FMT products frozen at -80°C for six months. All canine FMT products exhibited a significant decrease in overall bacterial viability at the six-month timepoint. Feline FMT products frozen with 10% glycerol or 25% M:D did not exhibit a significant decrease after six months of storage at - 80°C. Open circles represent individual replicates. The dashed line represents the limit of detection for bacterial growth using culture-based techniques. Significance testing was performed using the Wilcoxon matched pairs signed rank test comparing total CFU/gram at baseline and 6 months for canine and feline FMT products. Significance was defined as *p* < 0.05 for all pairwise analyses. Abbreviations: Fel, feline; Gly, gylcerol; K9, canine; MD, maltodextrin and trehalose.

As expected, at baseline, canine and feline raw fresh feces yielded significantly more CFUs/gram than 10% glycerol or 25% M:D FMT products (Kruskal-Wallis; dogs and cats, *p* < .01). For canine FMT products there was a significant decrease in bacterial viability of all frozen FMT products at the 6-month timepoint (Fig 7). Cryopreserved 10% glycerol FMT slurry had the highest mean CFUs/gram at the 6-month timepoint, however this was not significantly different compared to raw or 25% M:D (Kruskal-Wallis; p > 0.99).

In contrast with dogs, feline FMT frozen with 25% M:D did not exhibit significantly decreased CFUs/mL at the 6-month timepoint (Fig 7). CFU/g in feline FMT products at the 6-month timepoint were significantly higher in cryopreserved FMT products compared with stored raw frozen feline feces (Kruskal-Wallis; *p* < .01).

**Fig 8:**
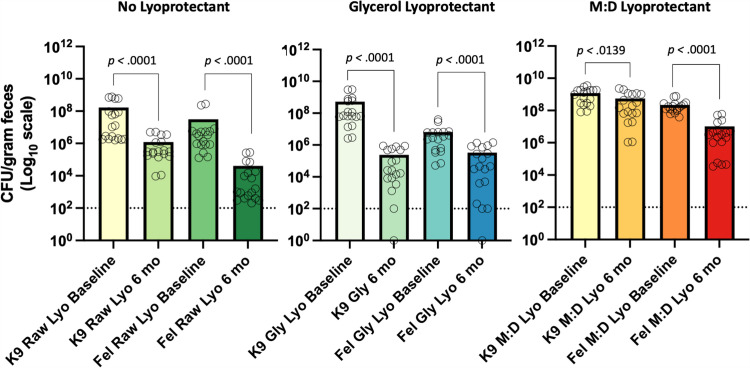
The long-term *in vitro* surrogate bacterial viability of lyophilized canine and feline FMT products stored at room temperature. In dogs and cats, lyophilization with M:D improves overall surrogate microbial viability compared to other lyopreservatives. Open circles represent individual replicates. The dashed line represents the limit of detection for bacterial growth using culture-based techniques. Significance testing was performed using the Wilcoxon matched pairs signed rank test comparing total CFU/gram at baseline and 6 months for canine and feline FMT products. Significance was defined as *p* < 0.05 for all pairwise analyses. Abbreviations: Fel, feline; Gly, gylcerol; K9, canine; lyo; lyophilized; MD, maltodextrin and trehalose.

Following 6 months of storage at room temperature, all lyophilized FMT products, regardless of species, showed a significant decrease in total CFUs/gram (Fig 8). Notably, 25% M:D yielded greater CFUs/gram compared to other lyopreservatives both at baseline (Kruskal-Wallis; dogs, *p* < .01; cats, *p* < .01) and at the 6-month timepoint (Kruskal-Wallis; dogs, *p* < .01; cats, *p* < .01) (Fig 8). Morover, lyophilized 25% M:D FMT products exhibited the smallest median log_10_ drop in bacterial viability (0.82), followed by raw (1.57), then 10% glycerol (3.42).

## 4. Discussion

Therapeutic manipulation of the intestinal microbiome has gained popularity in human and veterinary medicine; however, it remains underutilized in both fields. This study used amplicon sequence analysis to assess the microbial composition of fecal material throughout detailed FMT processing protocols (protocols available on protocols.io; overview Fig 1), with emphasis on initial (pooled raw feces; Step #2) and final steps (final FMT product; Step #7). The impact of centrifugation steps on processing FMT products was also evaluated. Bacterial viability of FMT products were evaluated using selective culture-dependent techniques in aerobic and anaerobic environments throughout a 2-week to 6-month timeframe for various FMT products in order to detect surrogate culturable bacterial members of the canine and feline gut microbiota.

As expected, beta diversity analysis showed that each canine donor has a distinct microbial community structure. This corroborates findings from previous studies, which determined that the intestinal microbial community varies between healthy dogs and are analogous to a unique fingerprint [33–35]. In canine donors, Bacillota was the most abundant phylum, followed by Actinomycetota and Bacteroidota in four and two donors, respectively. In one canine donor, Pseudomonadota was the second most abundant phylum, which is an unexpected finding in a clinically healthy and thoroughly screened fecal donor. While Pseudomonadota is a normal inhabitant of intestine, it typically constitutes a small fraction of the gut microbiota[3,4,33] and increased relative abundance has been associated with raw food diets[2,36–38] and dysbiosis[3,39]. Owners are instructed to inform the fecal bank of any dietary changes or indiscretion; however, in this case, we suspect one of these circumstances may have occurred without the owner’s knowledge, leading to an idiopathic and temporary shift in the donor’s microbial community.

In canine donors 1-4, certain microbial families, including *Enterobacteriaceae*, *Fusobacteriaceae*, and *Clostridiaceae* have a relative abundance of < 1% in homogenized pooled raw feces, but have a > 1% abundance in final FMT products (Fig 2C). Similar changes in *Enterobabcteriaceae* and *Fusobacteriaceae* were also observed in canine donors 5 – 7. Importantly, the change in relative abundance is compositional and is indicative only of the presence of DNA and not the viability of the microbe. Given the functional redundancy across microbes within the gut, the biological relevance of gain or loss of specific families is unknown. Though sterile equipment is used for fecal processing, the idiosyncratic shifts in the relative abundance of several microbial families may indicate environmental contribution of spores and/or vegetative cells. Feces are kept at 4°C throughout the standardized FMT processing protocol to reduce proliferation of microbes. However, the expansion of microbes such as *Enterobacteriaceae* and other facultative anaerobes could represent division of vegetative cells throughout the standardized FMT processing protocol. Though idiosyncratic shifts in relative abundance are observed in specific microbial groups, the overall microbial community structure and alpha diversity were not significantly impacted by processing and lyophilization (Figs 2-4, and Table 2). This was somewhat surprising, given the physical alteration of feces throughout these procedures (i.e., removing undigested material, concentration fecal microbes with double centrifugation, formation of a liquid slurry, and sublimation).

Centrifugation of FMT slurries has been proposed as a standardized processing technique in human medicine[11], but is not universally implemented in human or veterinary fecal repositories. While the microbial community structure is largely unaffected by the different processing techniques, FMT frozen at -80°C for two weeks without centrifugation shows significantly lower concentrations of CFU/g compared to both unprocessed and double-centrifuged FMT products (Fig 5, Table 3). The biological relevance of this remains unclear, as there was considerable overlap in the CFU/g range across these frozen FMT products. Further, there was no significant difference in overall surrogate microbial viability between freshly processed uncentrifuged and centrifuged FMT products (Fig 5, Table 3). This finding, combined with the similar overall CFU/g observed in frozen products, suggests that uncentrifuged FMT may be a practical alternative for veterinarians who do not have access to a large centrifuge, but wish to produce FMT in-house. Of note, double centrifugation techniques provide an additional benefit or reducing the volume of the original feces by approximately half the starting weight. This is advantageous in companion animal medicine because of the variability of animal size and dosing requirements.

As expected in both short-term and long-term storage, freshly voided feces consistently exhibited higher CFU/g compared to frozen and lyophilized FMT products; however, fresh feces may not always be available for immediate use and non-filtered FMT products often contain undigested material. In the author’s experience, this undigested material makes encapsulation challenging and may clog small bore catheters, making this option impractical for many veterinarians. Thus, we sought to determine the impact of processing technique on the surrogate microbial viability of canine and feline FMT products stored over a 6-month period. When frozen at -80°C, all canine products had significantly reduced viability after six months of storage; however, feces frozen without the addition of a cryopreservative suffered a greater loss of bacterial viability compared to FMT products processed with cryopreservatives (Fig 7). In contrast with dogs, feline FMT products frozen with 25% M:D did not exhibit a significant decrease in microbial viability after six months of storage, implying that the optimal cryopreservative varies between species (Fig 7). Anecdotally, bacteria isolated from cats are more difficult to culture and typically do not reach the same CFUs/mL as bacteria isolated from dogs. Thus, the addition of a cryopreservative in feline feces is particularly important to maximize revivification potential if freezing feline FMT products.

**Fig 9:**
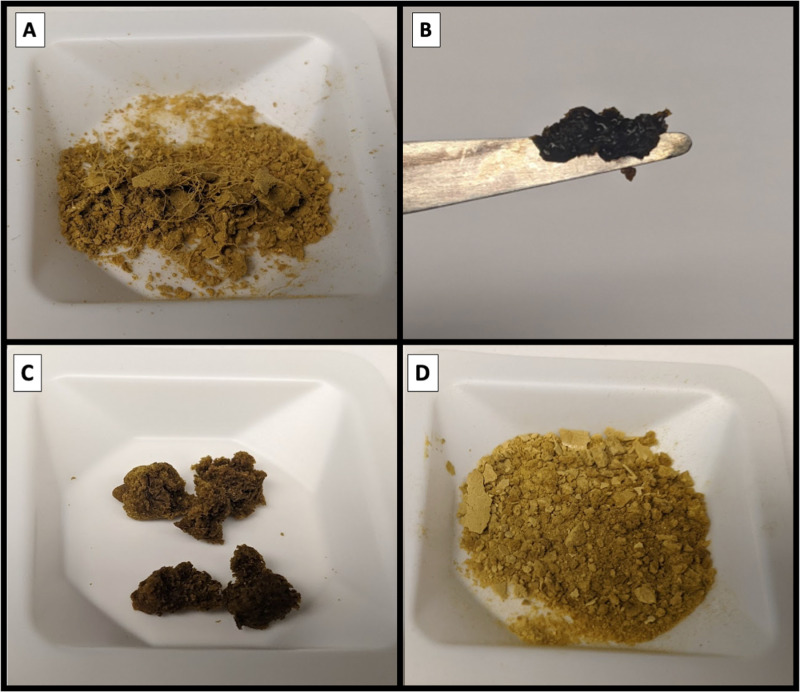
The impact of cryopreservative and centrifugation on the texture and consistency of four lyophilized FMT products. A) Unprocessed (raw) feces are unfiltered and contain hair and plant material; B) Uncentrifuged FMT product with 10% glycerol is extremely sticky and viscous, making it impractical for encapsulation. C) Double centrifuged FMT product with 10% glycerol is incompletely dry and tacky, making encapsulation subjectively difficult. D) Double centrifuged FMT product with 25% maltodextrin and trehalose (M:D) yields a homogeneous and completely dry product that is easily ground to a fine powder, improving the practicality for use in oral capsules.

All lyophilized products, regardless of cryopreservative, showed significantly reduced microbial viability after six months of storage at room temperature (Fig 8). However, in both dogs and cats, FMT preserved with M:D exhibited the highest median CFU/g at baseline and at six months, and had the lowest overall log_10_ drop in viability (Fig 8). M:D dissolved in saline produces a homogeneously viscous solution, which reduces ice crystal formation and osmotic stress, thus protecting microbial viability during freezing[40]. Importantly, the lyophilized M:D FMT cake is completely dry and easily ground to a fine powder, which is important for encapsulation (Fig 9). In contrast, lyophilized 10% glycerol FMT products are incompletely dry and form a sticky cake, making them difficult to homogenize. This retained moisture likely contributes to microbial loss over time by allowing for continued metabolic activity.

The shelf-life of FMT products is improved when microbial viability can be maintained over long-term storage. This reduces the need for frequent fecal collection and processing, thus reducing time, labor, and monetary resources required to run a fecal bank. However, the biological relevance of microbial viability in veterinary FMT products remains unclear. Given the paucity of evidence in veterinary medicine, additional blinded, disease specific, and randomized clinical trials evaluating the clinical efficacy of lyophilized and frozen FMT products on microbial engraftment and clinical outcomes are required in companion animals.

## 5. Limitations

The limitations of this study include a small sample size. The data presented is intended to provide evidence to support the protocols listed herein. Microbiota analysis was first performed using pooled fecal material from four screened canine fecal donor dogs, each providing 3 to 6 batches of stool for analysis. A second set of three screened canine fecal donor donors were used to test microbial community structure and viability using four different cryopreservatives. Additionally, amplicon sequencing and viability studies were not performed on non-bacterial constituents of the microbiome, including eukaryotes, bacteriophages, and protozoa. Additionally, the impact of processing on bacterial function and assessment of specific metabolites, such as SCFAs and bile acids, were not evaluated. Further research is needed to determine the effect of fecal processing on other non-bacterial microbes and postbiotics within FMT products.

Assessment of bacterial viability in centrifuged and non-centrifuged products were performed on a single stool sample from three dogs; and thus, may not have been adequately powered to reach statistical significance. The uncultivability of most intestinal bacteria precludes assessment of viability across the entire microbial milieu. Thus, the total CFUs/mL yielded from each FMT product on selective agar represents a small fraction of potentially viable microbes, which herein are considered a surrogate for the overall microbial community. The clinical relevance of the CFU/g “dose” required to confer a benefit for the FMT recipient is unknown. Further research is needed to determine whether increased CFU/g translates to improved microbial engraftment and clinical benefit.

## 6. Conclusions

Despite the proven clinical efficacy of FMT for the treatment of parvoviral enteritis[41], canine acute idiopathic diarrhea[42], and chronic inflammatory enteropathy[43,44], many veterinarians remain unfamiliar with the benefits and practicality of FMT as a microbial therapeutic for their patients[45]. To address this, the Companion Animal FMT Consortium, a group of international experts who have clinical and research experience with FMT in dogs and cats, recently developed clinical guidelines aimed at making FMT more accessible to veterinarians[22]. These guidelines offer a comprehensive review of the current literature and provide evidence that, regardless of preparation or route of administration, FMT is typically well-tolerated with minimal adverse effects [21,22,42,46,47].

The indications for FMT in companion animals are expanding as our understanding of disease-specific dysbiosis grows and evidenced-based recommendations for addressing dysbiosis continues to evolve. The work presented herein shows that canine FMT products cluster by donor, rather than by processing step or cryopreservative, indicating that the overall microbial community is not significantly impacted by a double centrifugation processing technique.

Although FMT products produced without centrifugation maintain high viability and are less labor-intensive to manufacture, their CFUs/gram are consistently lower compared to centrifuged products. However, important to note, the clinical impact on engraftment and clinical outcome is unknown. Filtration of undigested material (i.e., hair, plant material, chewed plastic) results in a product that can be readily homogenized and easily administered via oral or rectal routes.

For canine FMT products, 10% glycerol was the most effective cryopreservative for maintaining microbial viability over six months of storage at -80°C. For feline FMT, 25% M:D was the most-effective cryopreservative throughout six months of storage at -80°C. Finally, the double centrifuged 25% M:D FMT product is practical for lyophilization as it yields a completely dry cake and has superior maintenance of overall microbial viability compared to other lyophilized FMT products. These processing techniques are feasible in a variety of clinical settings and help preserve a microbial community closely resembling the original composition of voided feces, which may contribute to more reliable treatment outcomes. Ultimately, the protocols presented herein provides clinicians with detailed methods for consistent FMT manufacturing in any practice setting, making FMT more accessible for companion animals.
